# Novel Mutations in the *SCNN1A* Gene Causing Pseudohypoaldosteronism Type 1

**DOI:** 10.1371/journal.pone.0065676

**Published:** 2013-06-06

**Authors:** Jian Wang, Tingting Yu, Lei Yin, Jing Li, Li Yu, Ye Shen, Yongguo Yu, Yongnian Shen, Qihua Fu

**Affiliations:** 1 Research Division of Birth Defects, Institute of Pediatric Translational Medicine, Shanghai Children’s Medical Center, Shanghai Jiaotong University School of Medicine, Shanghai, P. R. China; 2 Department of Laboratory Medicine, Boston Children’s Hospital, Boston, Massachusetts, United States of America; 3 Department of Pediatrics, Shanghai Children’s Medical Center, Shanghai Jiaotong University School of Medicine, Shanghai, P. R. China; Innsbruck Medical University, Austria

## Abstract

Pseudohypoaldosteronism type 1 (PHA1) is a rare inherited disease characterized by resistance to the actions of aldosterone. Mutations in the subunit genes *(SCNN1A, SCNN1B*, *SCNN1G)* of the epithelial sodium channel (ENaC) and the *NR3C2* gene encoding the mineralocorticoid receptor, result in systemic PHA1 and renal PHA1 respectively. Common clinical manifestations of PHA1 include salt wasting, hyperkalaemia, metabolic acidosis and elevated plasma aldosterone levels in the neonatal period. In this study, we describe the clinical and biochemical manifestations in two Chinese patients with systemic PHA1. Sequence analysis of the *SCNN1A* gene revealed a compound heterozygous mutation (c.1311delG and c.1439+1G>C) in one patient and a homozygous mutation (c.814_815insG) in another patient, all three variants are novel. Further analysis of the splicing pattern in a minigene construct showed that the c.1439+1G>C mutation can lead to the retainment of intron 9 as the 5′-donor splice site disappears during post-transcriptional processing of mRNA. In conclusion, our study identified three novel *SCNN1A* gene mutations in two Chinese patients with systemic PHA1.

## Introduction

Pseudohypoaldosteronism type 1 (PHA1) is a rare inherited disease characterized by resistance to the actions of aldosterone. It was first described in 1958 by Cheek and Perry [1], and common clinical manifestations include salt wasting, hyperkalaemia, metabolic acidosis and elevated plasma aldosterone levels in the neonatal period [2]. According to the clinical manifestations and Mendelian inheritance patterns, PHA1 can be classified as either Renal PHA1 (autosomal dominant, OMIM177735) or the more severe systemic PHA1 (autosomal recessive, OMIM264350) [2]. Renal PHA1 results from a defect in the tubular response to aldosterone caused by inactivating mutations in the *NR3C2* gene (4q31) encoding the mineralocorticoid receptor [3]. Systemic PHA1 is caused by mutations in the genes encoding the subunits of the epithelial sodium channel (ENaC): alpha subunit (*SCNN1A*; 12p13), beta subunit (*SCNN1B*; 16p12.2-p12.1), or gamma subunit (*SCNN1G*; 16p12) [3]. However, some special cases of PHA1 have been reported. Hubert et al. [4] described a newborn with severe recessive PHA1 caused by two heterozygous mutations in *NR3C2*. Dirlewanger et al. [5] also reported that a homozygous missense mutation in *SCNN1A* is responsible for a transient neonatal form of PHA1.

In the 55 years since PHA was first known in the 1950s, sufficient clinical descriptions have accumulated in the literatures. Still, the symptoms of PHA1 are easily confused with that of other endocrine disorders, especially if the pediatrician cannot get specific biochemical findings [6]. The symptoms of PHA1 are easily confused with the symptoms of congenital adrenal hyperplasia (CAH) associated with 21-hydroxylase deficiency or 3-beta-hydroxysteroid dehydrogenase deficiency; and the symptoms of hypoaldosteronism (HA) due to aldosterone deficiency, antenatal or infantile Bartter syndrome. In addition, respiratory tract infections associated with PHA1 may be mistaken for symptoms of cystic fibrosis [7]. Such confusion delays diagnosis when prompt treatment of PHA1 is essential, as death may occur as a result of salt depletion and high blood potassium level [8].

In this study, two pediatric patients who suffer from PHA1 were promptly diagnosed with molecular genetic tests and treated. Using molecular genetic tests, we identified three novel mutations in the *SCNN1A* gene. We further confirmed the pathogenicity of the c.1439+1G>C mutation by analyzing its effect on splicing in a minigene construct.

## Subjects and Methods

### Patient

#### Case 1

A 5-day-old female infant was admitted to the neonatal intensive care unit as a result of her refusal to be breastfeed, poor response, weak cries and cold extremities. Physical examination: Weight 3100 g (SDS = −0.56), Height 49.5 cm (SDS = −0.37), Heart rate 125/min, Respiratory rate 36/min, Blood pressure 71/32 mmHg, Rectal temperature (37°C), SpO_2_ 99%. Auscultation: no lung crackles and heart murmur. Palpation: the liver edge about 2.5 cm below the costal margin, spleen is not palpable. Skin color: mild jaundice. Genital examination: female characteristics. Two days later, symptoms of severe dehydration and vomiting appeared. Biochemical laboratory tests showed that she had severe hyperkalemia with K^+^10.1 mmol/L, Na^+^105 mmol/L, Cl^+^83 mmol/L, PH 7.31, BE(B) −10.8 mmol/L and the trans-tubular potassium gradient (TTKG) is 4. Hormone indicator is as follows: aldosterone 1.08 nmol/L (range 0.17–0.47), plasma renin activity 14.0 ug/L/h (range 0–15), ACTH 20.4 pmol/L, cortisol 687.2 nmol/L, testosterone 0.94 nmol/L, and 17-OHP 25 nmol/L.

PHA1 was initially suspected on the basis of her clinical features, and the infant was immediately given a supplement of 10%NaCl. The results of hormone levels and TTKG value tests provided further evidence of PHA1 and the clinical diagnosis was confirmed by molecular genetic testing. After increasing doses of oral 10%NaCl to 30 mEq/kg/d and doses of ion-exchange resins to 1g/kg/d, the serum potassium level was controlled at 4.5 to 5.5 mmol/L. Her development seemed normal at the last follow-up visit (26 months). At that time, her plasma concentration of aldosterone is 1.25 nmol/L and plasma renin activity is 1.67 ug/L/h. Weight is 12.3 kg (SDS = 0.11), Height is 85 cm (SDS = −0.02).

#### Case 2

A 2-months old female infant was referred to our hospital with main complaint of electrolyte imbalance since she was born. Physical examination: Weight 4750 g (SDS = −4.13), Height 51.1 cm (SDS = −3.17), Heart rate 117/min, Respiratory rate 33/min, Blood pressure 80/35 mmHg. Genital examination: female characteristics. Other examination results showed that she is without lung crackles or heart murmur, and has normal liver and spleen size. This patient was born after 38 weeks of pregnancy. She weighed 2900 g (SDS = −0.65) at birth. At seven days of age, She developed signs of hyponatremia, hyperkalaemia and metabolic acidosis. She had initially been diagnosed with CAH in another hospital on account of her clinical presentation. However, 9α-fluorohydrocortisone (9α-FHC) supplement therapy was ineffective. There was no history of parental consanguinity. Laboratory test results are as follows: K^+^6.1 mmol/L, Na^+^125 mmol/L, Cl^+^92 mmol/L, PH 7.34, BE(B) −6.8 mmol/L and the TTKG is 7. Hormone indicator is as follows: aldosterone 0.58 nmol/L (range 0.17–0.47), plasma renin activity 10.1 ug/L/h (range 0–15), ACTH 4.5 pmol/L, cortisol 460.9 nmol/L, testosterone 0.42 nmol/L, and 17-OHP 13 nmol/L.

After obtaining the molecular diagnostic result, the baby was definitively diagnosed with PHA1. The serum potassium level was controlled with an intake of 10%NaCl at 10 mEq/kg/d and ion-exchange resins at 0.6 g/kg/d. At her last follow up at 12 months, physical examination showed normal results and her serum electrolytes are stable. Her weight was 9.5 kg (SDS = −0.09), and her height was 73 cm (SDS = −0.21).

The study protocol was approved by the ethics committee of the SCMC, and informed consent was obtained from the family members and normal subjects.

### Analysis of the *NR3C2*, *SCNN1A*, *SCNN1B* and *SCNN1G* Gene

The genomic DNA of the patients and their parents was isolated from whole blood samples by proteinase K digestion and phenol/chloroform extraction. An additional group of 105 Chinese subjects with no history of PHA1 were recruited as controls to test whether the mutations found are common polymorphisms. The study protocol was approved by the ethics committee of the hospital, and informed consent was obtained from family members and normal subjects.

All of the exons and exon-intron boundaries of the *NR3C2* (GenBank NM_000901.2), *SCNN1A* (GenBank NM_001038.5), *SCNN1B* (GenBank NM_000336.2) and *SCNN1G* (GenBank NM_001039.3) gene were amplified by PCR (TaKaRa, Dalian, China) using primers listed in Table 1. Primers were designed using Primer 3 software (http://frodo.wi.mit.edu/primer3/). The amplified PCR products were purified from agarose gel using QIAquick Gel Extraction Kit (Qiagen, Hilden, Germany) and sequenced via the ABI3730XL sequencer (Applied Biosystems, CA, USA).

**Table 1:** Primers for Amplify Exons and the Boundary Sequences of *SCNN1A*, *SCNN1B*, *SCNN1G* and *NR3C2.*

Gene	Exon	Primer Sequence (5'→3')		Product (bp)
		Forward	Reverse	
*SCNN1A*	1	tccttcgctgtccctctcta	ctcagctcctgcctctcact	739
	2	agggaggagtgggagaatgt	agctggaggctcctcatttt	791
	3	ctcctgcctctctccttcaa	tgggcaccaagaggtgttat	721
	4	tcctcagaaccccagatcac	cttccctcagatccagcagt	504
	5, 6	gctctgaaaggcacaagtcc	ctgctcctgaagacctccac	860
	7, 8	ggtggctggaagcatgtatt	gggaaactgacagaggcaga	723
	9∼12	tgggtgtggggtagagaaag	taagacccccagagcatcac	785
	13	ggagacagcttggtgaggag	cccttggttgtgttttgtcc	839
*SCNN1B*	1	ggtagcgcccagtaagctc	ctcccgtgggaaactgag	330
	2	ggagggtaaagagggaggaa	ggaaggaaaggaaggaaagg	537
	3	accttccgccatgattgtaa	ccccagcgagactcaaatta	581
	4	ttgagcatgtgtgagcatga	acaccgaggcacagaaaact	501
	5	actctcttcccctgctttcc	ggtagcagccactcctcttg	434
	6, 7	agtgggtagtggggtctcct	aaagtgactggtcccacagg	813
	8	tgtagctgcagccagtcatc	gtttcaagcccatgcttcat	514
	9,10	acctcctcctgccacctaac	cccacatcttatgcccagac	418
	11	ccttcctcccctagaacagc	cagtgacagagggaagcaca	474
	12,13	ctgtttggaagggggataca	ctttggagagggcaccatac	849
*SCNN1G*	1	agggggcgttgtgaagtc	ctctgagtggctctcgactg	462
	2	ggggccgtaagagaagtagg	ggctgtttaccagcgttagc	751
	3	ccacaggaagtcacacatgg	aggggctagtggtcaaggag	612
	4	ccaacctgttcccctgagta	accttttgctcccaagacct	413
	5	gagcaagatggggaaaatga	gatgtccctgtcgctctctc	483
	6	gcagtgggagaggtggttta	ggaccatgttcccttttgaa	358
	7	ccacagtaccaggcacctaa	aactgcagaggactggaacc	443
	8	cataaggggcaggttcatgt	atccaccgttcctacctcct	467
	9∼11	tggtagaaagtgggaggagaaa	gtggtgggaagagacagagg	650
	12	ttggggagcagttcttgagt	gcgggcaatgatagagaaga	721
	13	atcagggttcctgtgtgagg	tcctcactctggccttcact	762
*NR3C2*	1	cgggatagcaacctgaactt	agggggagaaaagtggaaaa	569
	2A	tgttctgacatctcgacaagc	aaacagacgggcttttctca	784
	2B	gaacacgcccttgagatcat	gccatccataaatggaaacg	895
	2C	ccagaaccagatggagcttt	atgccccttcaaaatcaatg	786
	3	tagcattgctccactcatcg	tttgtggaaaatctccaggtg	491
	4	cagctgcattaagctgacca	agcaaactcaggctcgaaaa	530
	5	tggaataaacggtcatgttcct	taaccctgcattctcggaag	680
	6	ggctgtttggggttgactta	tcttcccaattggagtcgat	577
	7	ggcccagcagtattggtcta	tgagtggttggatggatgaa	569
	8	cctgccaagatgctaaagttg	tcttggcccatcctgtatgt	441
	9	ccaaagtcagaaggcagagg	aaatggacgctaacgagtgtg	830

### Minigene Construction and Transfection into HEK 293 Cells

The splice region (c.1439+1, intron 9) of *SCNN1A* gene was amplified with specific primer pair (5′-CCCaagcttGTAGAGAAAGCTGAGGTGCC-3′, and 3′-GACGCCGATTCAGAGAAAAAcctaggCTG-5′) using PrimeSTAR HS DNA Polymerase (TaKaRa, Dalian, China). The forward and reverse primer sequences are located in intron 8 and in 3′UTR, respectively. The length of the PCR product is 1,863 bp. The restriction enzyme sites *Hind*III and *Bam*HI were inserted into the primer sequences to enable directional cloning. DNA from a normal individual was used as control. Minigene amplification products were purified, digested, and ligated into the pcDNA3.1/Myc-His B vector (Invitrogen, CA, USA) according to the protocol of the manufacturer. The constructions were used to transform *E. coli DH5α* competent cells, and putative recombinant colonies were selected to verify the presence of the correct mutation by sequencing recovered plasmids. The recombinant plasmids with either wild-type or mutant *SCNN1A* were transfected into HEK 293 cells. Blank vector was used as a control.

### RNA Extraction, cDNA Synthesis, and Splice Sites Analyzes

Thirty-six hours after transfection, total RNA from the HEK 293 cells was extracted with an RNeasy Mini Kit (Qiagen, Hilden, Germany). cDNA chains were obtained by reverse transcription (TaKaRa, Dalian, China) and target fragment was amplified with another primer pair (5′-ttgacttctcctcagaccacc-3′, and 5′-agggtgaccatcgtgacagag-3′). The forward and reverse primer sequences are located in exon 9 and at the junction between exon 10 and 11, respectively. The expected length of the PCR product is 256 bp. Then, PCR products were analyzed by running in 2% agarose gel stained with ethidium bromide and sequenced.

## Results

### Mutational Analysis

**Figure 1:**
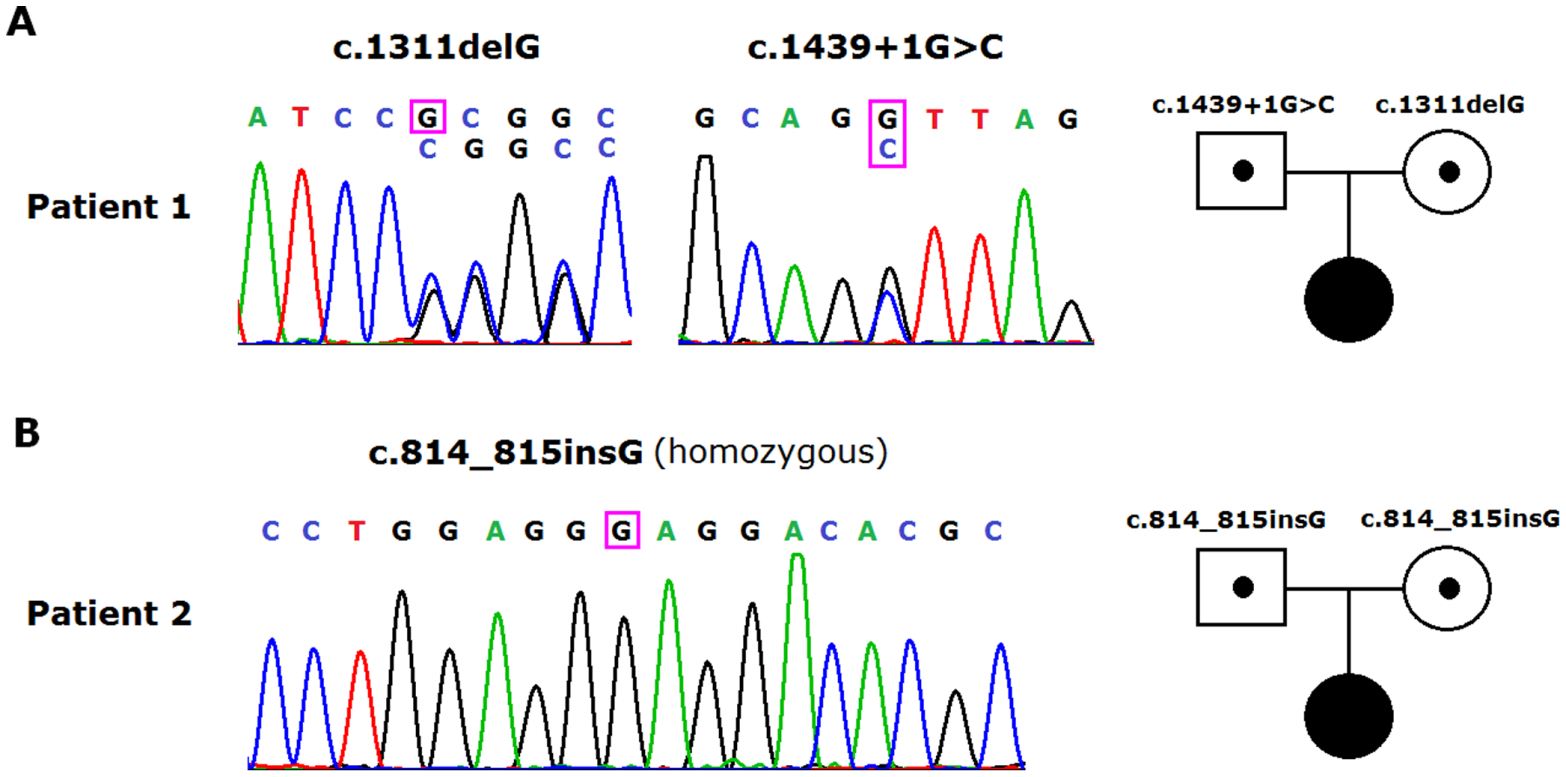
Three novel mutations were identified in the *SCNN1A* gene. (A) Sequences showing a compound heterozygous mutations (c.1311delG in exon 8 c.1439+1G>C in intron 9) in PHA1 patient of case 1. (B) Sequences showing a homozygous mutation (c.814_815insG in exon 4) in PHA1 patient of case 2.

In case 1, the patient and her parents were genotyped by direct nucleotide sequencing. We found that the patient had compound heterozygous mutations in the *SCNN1A* gene. One mutation was a deletion of a single base “guanine” (c.1311delG) in exon 8, which resulted in a frameshift leading to a premature stop codon (p.Arg438GlyfsX43). The other mutation was a transversion of guanine to cytosine (c.1439+1G>C) in the first base of the 5′-donor splice site of intron 9. This mutation occurs at a highly conserved “AG-gt” sequence of the exon-intron boundary region. The predicted effect of this G>C mutation is a disruption normal splicing, possibly resulting in the retainment of intron 9 of the *SCNN1A* gene. No other mutations were found in the entire exons and the exon-intron boundaries of the patient's *NR3C2, SCNN1B* and *SCNN1G* gene. The patient’s mother was heterozygous for the c.1311delG mutation, and her father was heterozygous for the c.1439+1G>C mutation. No such mutations were observed in the 105 unrelated healthy individuals. Consequently, it was concluded that the patient is a novel compound heterozygous individual who inherited the maternal c.1311delG mutation and the paternal c.1439+1G>C mutation (Fig. 1A).

In case 2, direct sequencing of the entire *SCNN1A* gene in the patient revealed a homozygous insertion of one “G” in exon 4 (c.814_815insG). This frameshift mutation results in a premature stop codon (p.Glu272GlyfsX38). The mutation was also found in heterozygous state in both of her parents. DNA samples from 105 unrelated healthy individuals were subsequently tested for this mutation, and it was not found in these individuals. Therefore, we concluded that the patient is a novel homozygous individual who inherited a copy of the c.814_815insG mutation from each of her heterozygous parents (Fig. 1B).

### *In vitro* Splicing Assays to the c.1439+1G>C Mutation

**Figure 2:**
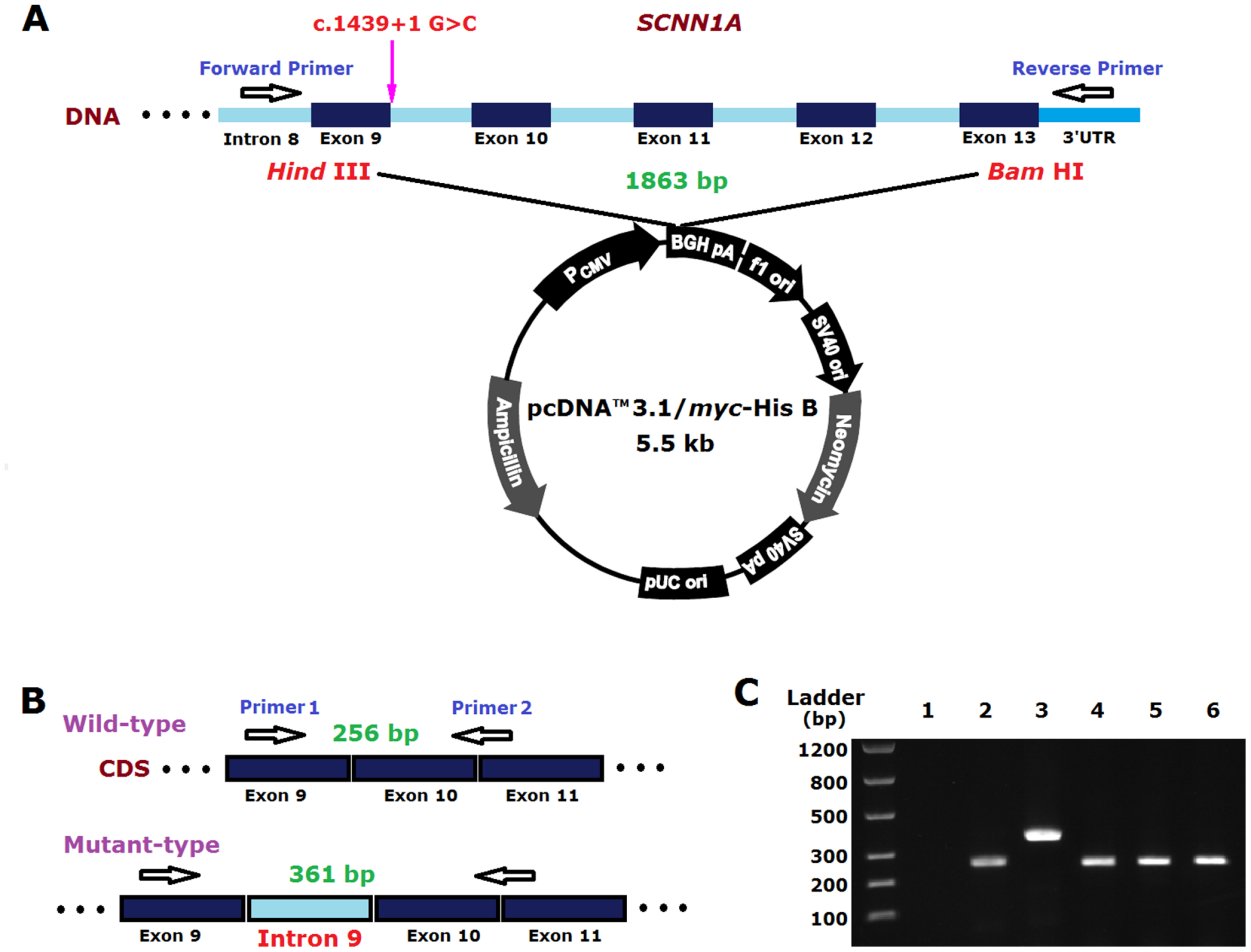
In vitro splicing assay of the c.1439+1G>C mutation. (A) The splice region (c.1439+1, intron 9) of *SCNN1A* gene was amplified and products were ligated into the pcDNA3.1/Myc-His B vector. (B) RT-PCR of HEK293 cells transfected with either wild-type or mutant *SCNN1A.* Minigenes showed that the mutation c.1439+1G>C was sufficient to produce a longer band. (C) Lane 1: Empty pcDNA3.1 vector; Lane 2: wild-type *SCNN1A* (256 bp); Lane 3: c.1439+1G>C mutant (361 bp); Lanes 4, 5, and 6: *GAPDH* used as control (245 bp).

To assess the impact of the splice-site mutation, a minigene construct was generated and tested in transiently transfected cultured cells. HEK 293 cells were transfected with the mutant *SCNN1A*, wild type *SCNN1A*, or empty pcDNA3.1 vector (negative control). Three independent replicates were tested, and *GAPDH* was used as a control for RNA quality and quantity. A difference in the size of the cDNA fragment was observed between wild type and mutated *SCNN1A.* The wild-type *SCNN1A* resulted in a single band at 256 bp, whereas the mutated construct resulted in a longer band at 361 bp. Sequencing confirmed that RNA resulted from mutant *SCNN1A* construct contained the 105 nucleotides that constitute intron 9. The loss of the 5′-donor splice site at the exon-intron boundary resulted in the retainment of intron 9 in the RNA (Fig. 2).

## Discussion

Pediatricians should consider the possibility of PHA1 when encountering infants with unexplained hyponatremia, hyperkalemia and metabolic acidosis [2]. PHA1 diagnosis is based on elevated plasma aldosterone and renin levels, especially when high-dose mineralocorticoid treatment cannot remedy the imbalance of potassium and salt. Schweiger et al. [9] suggested that TTKG index may be helpful in the acute setting to determine whether mineralocorticoid deficiency or resistance is leading to hyperkalemia. A typical TTKG value in a normal person on a normal diet is 8–9 [10]. During hyperkalemia or high potassium intake, more potassium should be excreted in the urine and the TTKG should be above 10. If the TTKG is low during hyperkalemia, and this is also accompanied by hyponatremia and increased urine sodium excretion, the presence of mineralocorticoid deficiency or resistance should be considered. Belot et al. [11] reported that there are two clinical manifestations in patients with systemic PHA1, including life-threatening weight loss and early cutaneous involvement. The dermatitis may be due to high salt concentration in sweat. It is recommended for weight changes to be monitored closely, and for sweat test (which measures the concentration of chloride) to be administered for early diagnosis [11]. In addition, detailed family history is often crucial.

In our case, both patients had signs of salt loss soon after birth, including hyponatremia, hyperkalemia, and metabolic acidosis. We considered the possibility of “PHA1” in accordance with their hormone levels and TTKG results. Molecular genetic tests were performed on each patient and her family in order to confirm the clinical diagnosis. All of the exons and exon-intron boundaries of the *NR3C2*, *SCNN1A*, *SCNN1B* and *SCNN1G* genes were sequenced by Sanger method. The results showed that the two patients had novel mutations in the *SCNN1A* gene respectively.

Since the first identification of mutations in the Saudi and Iranian Jewish kindred of PHA1 [12], nearly 25 kinds of mutations have been identified in *SCNN1A* gene, including missense/nonsense mutations, splicing mutations, small insertions/deletions, and gross deletions [7], [13]–[16]. However, no mutations have been reported in the Chinese population. We have successfully identified two novel frameshift mutations (c.1311delG and c.814_815insG) and a novel splicing mutation (c.1439+1G>C) in the *SCNN1A* gene in two Chinese patients with systemic PHA1.

The *SCNN1A* gene contains 13 exons and encodes the alpha subunit of the ENaC, which is expressed in the distal nephron and regulated by aldosterone [17]. The ENaC is the rate-limiting step for sodium reabsorption in the apical membrane of epithelia [18]. It plays a major role in the Na+- and K+-ion homeostasis, and has a high affinity for the potassium-sparing diuretics amiloride and triamterene. In our study, the frameshift mutations c.1311delG and c.814_815insG induce premature termination of mRNA translation and cause loss of the C-terminal domain (extracellular, helical and cytoplasmic) of the protein. The splicing site mutation (c.1439+1G>C) can lead to the retention of intron 9 as the 5′-donor splice site disappears during post-transcriptional processing of mRNA. All of these novel mutations are thought to result in an absence of a functional αENaC subunit, and constitute the molecular basis for systemic PHA1 phenotype.

At present, the main treatment for PHA1 is the intake of high doses of sodium together with ion exchange resins and dietary manipulations to reduce potassium levels [8]. During the course of treatment, clinicians must gradually adjust the dose according to the serum electrolytes and hormone levels [10], [11]. When treatment is effective, PHA1 patients will be able to maintain the right balance of electrolytes and resume normal growth and development; their plasma concentration of aldosterone and renin activity will be reduced or return to normal [8]. In order to reach such successful treatment outcome, patients must take medicine on time, maintain the right dosage, avoid high potassium foods and closely observe signs and symptoms of PHA1 [8]. In our case, patient from case 1 was hospitalized again after four months due to hyperkalemia caused by diarrhea. Because both patients suffer from the systemic form of PHA1, both may need lifelong therapy. Because PHA1 is rare, the relationship between genotype and phenotype is not yet clear as a result of lack of sufficient cases. However, our study has brought us a step closer to understanding this relationship, and may ultimately contribute to the goal of finding a simpler and more effective treatment for these patients.

### Conclusions

We identified three novel *SCNN1A* gene mutations in two Chinese patients with systemic PHA1. The c.1311delG mutation and the c.814_815insG mutation are both frameshift mutations that lead to premature stop codon. As demonstrated by the functional analysis using an *in vitro* minigene system, the third c.1439+1G>C mutation leads to aberrant splicing, in which intron 9 is retains as the 5′-donor splice site of intron 9 disappeared.
